# Massive data analyses show negative impact of type 1 and 2 diabetes on the outcome of periodontal treatment

**DOI:** 10.1007/s00784-020-03512-0

**Published:** 2020-08-20

**Authors:** Michael Raedel, Barbara Noack, Heinz-Werner Priess, Steffen Bohm, Michael H. Walter

**Affiliations:** 1 Prosthodontics, Carl Gustav Carus Faculty of Medicine, TU Dresden, Fetscherstr. 74, 01307 Dresden, Germany; 2 Periodontics, Carl Gustav Carus Faculty of Medicine, TU Dresden, Dresden, Germany; 3 AGENON GmbH, Berlin, Germany

**Keywords:** Periodontics, Treatment outcome, Re-intervention, Extraction, Diabetes mellitus

## Abstract

**Objectives:**

The aim was to evaluate the impact of diabetes on the outcome of periodontal treatment based on massive data analyses.

**Materials and methods:**

Data originated from the database of a major German National Health Insurance. Patients who underwent periodontal treatment were allocated to four groups according to their medical condition: type 1 diabetes (D1), type 2 diabetes with the intake of oral anti-diabetics (D2M), type 2 diabetes without the intake of oral anti-diabetics (D2), and a control group without diabetes (ND). Four-year Kaplan-Meier survival analyses on the patient level and multivariate regression analyses were conducted for tooth extraction.

**Results:**

Of 415,718 patients, 4139 matched the criteria for D1, 22,430 for D2M, and 23,576 for D2. At 4 years, the cumulative survival rate (no extraction) was 51.7% in the D1 group, 54.0% in the D2M group, and 57.7% in the D2 group. The ND control group had a significantly higher survival rate of 65.9% (*P* < 0.0001). In the multivariate analyses, both diabetes types were significantly associated with further tooth loss after periodontal treatment.

**Conclusions:**

The diagnosis of diabetes type 1 or 2 seems to be associated with a higher risk of tooth loss after periodontal treatment.

**Clinical relevance:**

The long-term prognosis of teeth in diabetes patients should be judged carefully.

## Introduction

Periodontitis is one of the most prevalent oral diseases worldwide [1–6]. It is the result of bacterially induced inflammation leading to the gradual destruction of periodontal tissues and possibly also to tooth loss [7, 8]. The pathogenesis of periodontitis is multifactorial. The host response to pathogenic bacteria is influenced by behavioral, environmental, genetic, and epigenetic risk factors [9]. Over the decades, a widely accepted concept for treating periodontitis has been established. After a so-called initial active periodontal treatment including subgingival biofilm removal, periodontal maintenance procedures should support patients in maintaining periodontal treatment outcomes [10]. Further attachment loss and tooth loss after periodontal treatment is associated with a number of tooth and patient-related factors [11–14].

The World Health Organization (WHO) estimates that, in 2014, 8.5% of the world population suffered from diabetes [15]. Diabetes and periodontitis are both chronic inflammatory diseases sharing some common pathomechanisms, such as hyper-inflammation and impaired immunological host responses. The current model of a bidirectional relationship of both diseases is well established [16–19]. In that sense, diabetes is one of the main risk factors for periodontitis, and periodontitis is now considered as a further complication of diabetes [20]. The impact of glycemic control on periodontitis progression and tooth loss seems to be dose depending [21]. Contrary to these findings, the authors of a clinical study from the USA conclude that neither the HbA1c value nor the duration of diabetes was associated with periodontal treatment outcome after 6 months [22]. A recent review pointed in the same direction [16]. In patients with chronic periodontitis and diabetes, treatment outcome regarding pocket depth reduction and attachment gain seems to be independent of metabolic control in the short term. These treatment outcomes in patients with diabetes were not inferior to those in non-diabetes patients. However, data on the magnitude of the impact of diabetes on mid-term and long-term treatment outcomes are sparse. Diabetic patients with poor metabolic control may tend to have a less favorable long-term outcome [23]. In addition, only few studies reported an increased risk for tooth loss after active periodontal treatment [24, 25].

Most of the aforementioned studies were standardized trials with smaller study populations. Results from population-based studies and practice-based research are barely available. The aim of this study was to examine associations between periodontitis treatment outcomes and both types of diabetes on a population level based on massive data. It was hypothesized that the outcome of periodontal treatment is poorer in diabetes type 1 and 2 patients compared with patients without diabetes.

## Materials and methods

This study based on claims data from a major German National Health Insurance company (BARMER, Berlin, Germany). These routine data were not collected for scientific purposes. In the context of an annual oral health care report, the study team had access to the company’s data warehouse [26]. The responsible local ethics board confirmed ethical approval (EK 288072015).

The basic unit of information within the database was a fee code. This fee code represented a provided service, for example, periodontal treatment and tooth extraction. Diagnoses were not available. In terms of the patients’ medical condition, diagnoses were accessible according to the International Statistical Classification of Diseases and Related Health Problems (ICD-10) and prescriptions for medications. For basic dental treatments like extractions, patient-specific fee codes and dates allowed for tracing clinical courses on a day count basis.

The observation period was between January 1, 2012, and December 31, 2015. All members of the insurance company receiving a periodontal treatment within the whole 4-year observation period and staying member of the insurance thereafter entered the analysis. The billing date was considered indicating a concluded periodontal treatment. Patients were allocated to four groups (Table 1).

**Table 1:** Study and control groups

Group	Description
Study group D1	Patients with type 1 diabetes documented diagnosis at least twice a year
Study group D2M	Patients with type 2 diabetes documented diagnosis at least twice a year and prescription of oral anti-diabetics
Study group D2	Patients with type 2 diabetes documented diagnosis at least twice a year without prescription of oral anti-diabetics
Control group ND	All other patients

Kaplan-Meier survival analyses on a day count basis were conducted on a patient level for the primary outcome “first extraction after periodontal treatment.” Extractions within 60 days after concluded periodontal treatment were regarded as being connected to the periodontal treatment and therefore not counted. Differences between the groups were tested for significance with the log-rank test (*P* < 0.05).

Additionally, two independent multivariate analyses were carried out. In a multivariate Cox regression analysis for the dependent variable “first extraction after periodontal treatment,” the following independent variables were included: age group, gender, the use of regular dental checkups at least once a year, the number of periodontally treated teeth, surgical procedures within the periodontal treatment, periodontal treatment of both one- and multi-rooted teeth, type 1 diabetes, and type 2 diabetes with medication. Dental checkups comprised no treatment.

Focusing on multiple target events, a weighted linear regression analysis was conducted for the dependent variable “extractions per year.” The regression was weighted according to the length of the observation period. The following independent variables were included in the regression model: gender, age, the use of dental checkups at least once a year, diabetes type 1, and diabetes type 2 with medication.

The software R (available from http://www.r-project.org) with the add-on package “survival” was used for statistical analyses.

## Results

A total of 415,718 patients underwent a periodontal treatment between January 2012 and December 2015. Within this sample, 4139 patients (1.00%) matched the criteria for type 1 diabetes, 22,430 patients (5.40%) for type 2 diabetes with medication, and 23,576 patients (5.43%) for type 2 diabetes without medication. They were allocated to the respective study groups. The remaining 365,573 patients (87.94%) who did not match any diabetes criteria formed the control group. The median age in all four groups ranged between 50 and 55 years of age (Table 2).

**Table 2:** Age distribution within the study population and the study groups

Age group (years)	Study population	Type 1 diabetes (D1)	Type 2 diabetes with medication (D2M)	Type 2 diabetes without medication (D2)	No diabetes (ND)
18–24	2,504	14	2	10	2478
25–34	21,723	129	86	142	21,366
35–44	47,190	270	519	768	45,633
45–54	109,454	716	2828	3407	102,503
55–64	114,739	1212	6801	6851	99,875
65–74	83,717	1203	8145	8017	66,352
75–84	33,630	574	3780	4024	25,252
85–94	2,740	21	269	356	2094
> 95	21	0	0	1	20

**Fig. 1:**
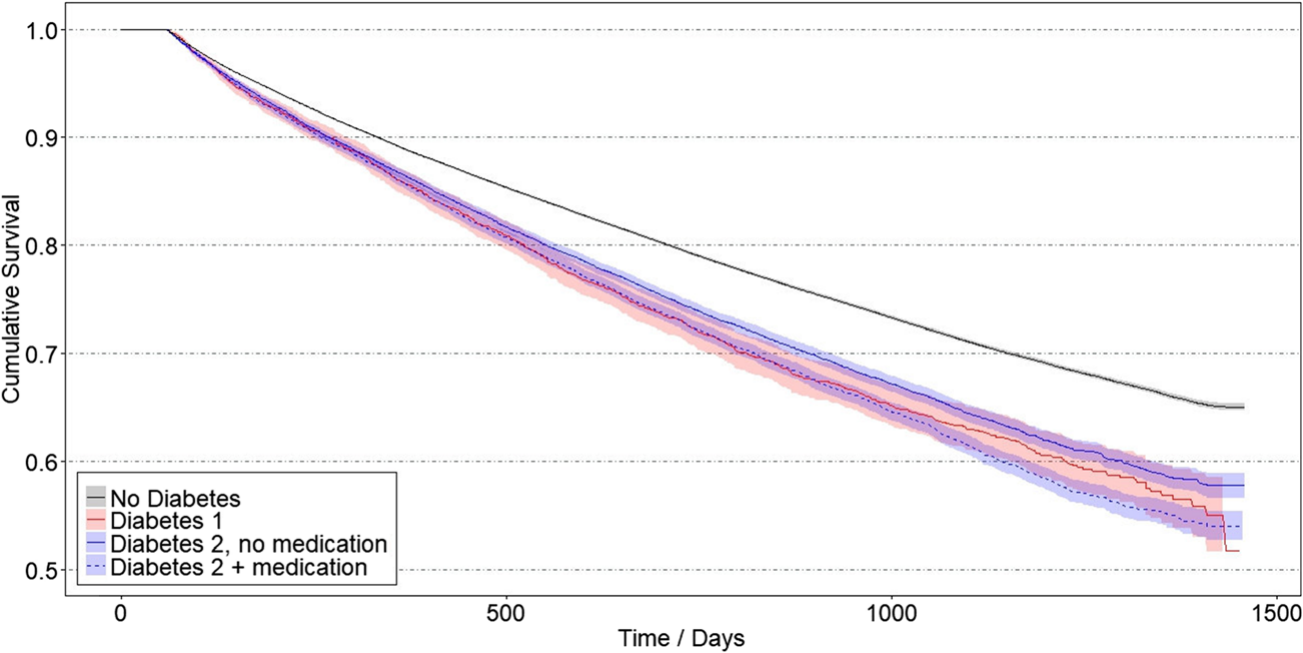
Kaplan-Meier survival functions with 95% confidence intervals for the target event extraction for study groups and control groups. Extractions within 60 days after treatment were not counted

The Kaplan-Meier survival estimation considers the number of event-free teeth at the time of a target event. The higher that number is, the narrower the confidence interval will be. Table 3 shows the decreasing numbers of teeth under risk at 1 year, 2 years, 3 years, and at the time of the last event within the groups. The 4-year survival rates for extraction were 51.7% for the type 1 diabetes group, 54.0% for the type 2 diabetes with medication group, 57.7% for the type 2 diabetes without medication group, and 65.0% for the non-diabetes control (Fig. 1). After the initial 60-day plateau where extractions were not counted, the survival curves followed a close to the linear course. The differences between the diabetes groups and the control group were highly significant with *P* < 0.0001.

**Table 3:** Number of teeth under risk after 1 year, 2 years, 3 years and at the time of the last event

Time	Type 1 diabetes 1 (D1)	Type 2 diabetes with medication (D2M)	Type 2 diabetes without medication (D2)	No diabetes (ND)
1 year	2775	14,676	16,658	252,620
2 years	1571	8088	10,102	150,064
3 years	682	3246	4551	65,881
Time of last event	17	268	417	1774

The results of the multivariate Cox regression analysis after periodontal treatment show significant associations of both diabetes types with the target event “first extraction” with hazard ratios above 1 (Table 4). Type 1 diabetes was associated with a 20% higher probability for tooth loss after periodontal treatment. Type 2 diabetes with medication was associated with a 16% higher probability. The more teeth were treated, the lower was the probability of extraction after periodontal treatment. Each additionally treated tooth lowered the probability by 2%. Surgical periodontal treatment increased the probability of 13%. The female gender slightly lowered the probability by 5%. With higher age, the probability of tooth loss increased by 15% per 10-year WHO age group. The fit of the Cox regression model was good with a concordance of 0.578 and a respective standard deviation of 0.001.

**Table 4:** Multivariate Cox regression analysis for the first extraction after periodontal treatment

Variable	Hazard ratio	95% confidence interval	*P* value
Type 1 diabetes	1.2018	1.1317–1.2762	< 0.0001
Type 2 diabetes with medication	1.1579	1.1270–1.1898	< 0.0001
At least one dental checkup per year	0.9815	0.9581–1.0054	> 0.05
Number of treated teeth (per tooth)	0.9786	0.9775–0.9797	< 0.0001
Surgical periodontal treatment involved	1.1274	1.0868–1.1696	< 0.0001
Both one- and multi-rooted teeth treated	1.5510	1.1126–2.1622	< 0.05
Female gender	0.9468	0.9338–0.9601	< 0.0001
Increasing age (per 10-year WHO age group)	1.1491	1.1429–1.1553	< 0.0001

The second, weighted linear regression model considered not only the first extraction but all extractions after periodontal treatment within the observation period (Table 5). For each patient, we counted all extractions in relation to the observation period after the initial 60 days. We calculated the number of extractions per patient and year and conducted a weighted linear regression analysis. The observation time was included as weight. An estimator 1 means that when controlling for all other effects, this variable additionally accounts for one extraction per year. The highest estimators in the model were found for both diabetes types. Type 1 diabetes showed a higher estimator than type 2 diabetes. The effect of yearly dental checkups was low. For gender, no effect was found. Per the higher WHO age group, 0.05 more teeth were extracted per year. The multiple *r*^2^ value and the adjusted *r*^2^ value were both 0.1279 meaning 13% of the variance for extractions was explained by the model. This indicated a high explanatory power because the analysis had been carried out on the individual level.

**Table 5:** Multivariate model including all extractions after periodontal treatment

Independent variable	Estimator	Standard error	*t* value	*P* value
Type 1 diabetes	0.0774	0.0093917	8.237	< 0.0001
Type 2 diabetes with medication	0.0547	0.0042511	12.857	< 0.0001
At least one dental checkup per year	− 0.0075	0.0019477	− 3.868	< 0.001
Female gender	0.0023	0.0018732	1.250	> 0.05
Increasing age (per 10-year WHO age group)	0.0464	0.0003874	119.748	< 0.0001

## Discussion

This study bases on more than 400,000 cases with a concluded periodontal treatment within a defined period of 4 years resulting in an observation period of 4 years at maximum. It reveals relationships between both diabetes types and extractions after periodontal treatment.

The study based on claims data and therefore has inevitable limitations. The database comprised the complete number of all treatments expressed through fee codes for 8.5 million members of the insurance company representing approximately 10% of the German population in December 2015. For the dental part, no further clinical information as findings and diagnoses including those related to periodontal disease and tooth extraction were available. Most striking is the lack of information about the disease severity that is not reflected by the fee codes. This specific limitation might have biased the results because of potential differences in severity between the groups. The same applies to other confounders such as smoking and compliance. An estimation of the extent of a possibly resulting bias cannot be based on data and would therefore be speculative. The period of 60 days after a concluded treatment in which we did not count extractions was somewhat arbitrary but considered justified. For the medical part, some clinical information was accessible through diagnoses and prescriptions. On the other hand, no information regarding the metabolic control of diabetes patients was available. Generally, claims data are prone to mistakes and errors. As a matter of course, we have no information about the quality of periodontal treatment. However, there is no reason for assuming quality-related differences between the groups. A number of limitations were similar to the ones discussed with other analyses of different dental treatment outcomes from this data source [27–31]. Irrespective of the weaknesses, the extremely high case numbers, the use of a hard outcome variable, and the closeness to reality are evident strengths. For this reason, the use of massive data resources for evaluating outcomes from general dental practice has great potential and can be expected to gain increasing future importance. Massive data analyses do not replace clinical studies but supplement them with results from a completely different perspective.

In view of the study design and the inherent limitations, the results require a careful and appropriate interpretation. From the data, no causal relationships can be derived. Among the claims data, periodontal treatment is clearly defined and easy to identify. For each treatment, there is information about the number of treated single-rooted and multi-rooted teeth, non-surgical and surgical (flap) interventions, and the billing date.

For diabetes, a diagnosis based on the ICD-10 coding is available. The prevalence of both diabetes types within the study population was 12.1%. This closely matches the prevalence of diabetes published by the International Diabetes Federation for Germany in 2017 being 12.2% [32]. Therefore, the study population is considered as being nearly representative of the German population. From previous analyses in German routine data, we know that there is a high probability that patients marked twice a year with the ICD-10 code for type 1 diabetes do actually have this condition. However, with type 2 diabetes, this probability might be lower. Therefore, the regular intake of oral anti-diabetics was selected as an additional inclusion criterion for the type 2 diabetes group. Nonetheless, we also reported a type 2 diabetes without medication group for comparison.

The question of whether periodontal treatment affects diabetes parameters is widely researched. There are numerous publications dealing with the effect of periodontal treatment on diabetes parameters [33–38]. While several intervention studies showed the effects of periodontal treatment on glycemic control, one large randomized controlled trial by Engebretson et al. [37] reported no such effect. This trial was accused of having serious shortcomings regarding study design, recruitment, and intervention outcomes. Periodontal treatment was suspected to have been below the expected standard of care [39]. The intervention in our study is a black box providing no quality indicators. However, we expect no substantial quality differences in the interventions for diabetes and non-diabetes patients on a population level. In consequence, we assume a still acceptable external validity in terms of the outcome differences. There is still no consensus about whether diabetes might have negatively affected the treatment outcome. In a recent review, the hypothesis has not been supported that type 2 diabetes with poor glycemic control could affect short-term outcomes after scaling and root planing. On the other hand, the authors concluded that during periodontal maintenance small effects of diabetes might often remain undetected [16]. Within the review, only six out of 17 retrospective studies analyzed diabetes effects [24, 40–44]. Only two of these revealed diabetes as a significant predictor for tooth loss [24, 41]. A population-based study using the Japanese database of health insurance claims showed more tooth loss in periodontally treated diabetes patients compared with non-diabetes patients [45]. Although these results point in the direction of our findings, a comparison with our data is compromised by the composition of the control group consisting of patients without diabetes but with acute upper respiratory inflammation.

Our results are rather straightforward. Whereas 65% of the non-diabetes patients showed no extractions up to 4 years after periodontal treatment, for those with diabetes diagnoses, this percentage was substantially lower with 51.7% for the D1 group, 54.0% for the D2M group, and 57.7% for the D2 group. Because of similar age distributions in the groups, we do not assume age-related effects. Although data on metabolic control are lacking, these differences between the diabetes groups might hint at a dose-response effect since metabolic control might decrease from D1 over D2M to D2.

The multivariate models support the results from the Kaplan-Meier analyses. They also show that other independent variables were not superimposing the association between diabetes and treatment outcome. As mentioned above, lacking information about findings and diagnoses are significant limitations. There still might be other unknown or inaccessible variables and factors (smoking, etc.). A major uncertainty about the validity of the more unfavorable outcomes in diabetes patients is the unknown stage of periodontitis at the time of treatment. If there was a difference in the stages in patients with and without diabetes (e.g., a greater severity in the diabetes groups), this would have biased the results. However, we do not have any respective information. What we can reliably say is that within the German health care system, the outcome in treated diabetes patients was poorer than in non-diabetes patients. As clearly pointed out before, the underlying causes cannot be derived from our data.

In general, treatment outcomes in clinical reality or on a population level are expected to be inferior to those under optimal conditions, for example, in specialized dental practices or within a well-designed clinical trial. This existence of an efficacy-effectiveness gap applies also to periodontal treatment and has been reported in a previous publication based on this data pool [31]. The external validity of the results is affected by the regulations of the German health care system. Treatment need is taken for granted in cases of probing depths of at least 4 mm. In this regard, substantial differences in other countries have to be assumed.

Summarizing, the study hypothesis can be confirmed. Actually, this is the first study revealing significant associations between both diabetes types and periodontal treatment outcomes based on massive data. Because of its much lower prevalence compared with type 2 diabetes, the effects of type 1 diabetes have been rarely studied before.

## Conclusion

The diagnosis of diabetes type 1 or 2 seems to be associated with a higher risk of tooth loss after periodontal treatment.
